# Association Between Metabolically Associated Steatotic Liver Disease (MASLD) and Cardiovascular Risk Scores in Urban Adults

**DOI:** 10.14789/ejmj.JMJ25-0042-OA

**Published:** 2026-01-21

**Authors:** FARIS HUSSAIN, KELVIN KANAYO NWABUEZE, FATIMA IJAZ, SONIA OUTALEB, MAHMUDUL HASAN NAHID, SYED ABID, MARIAM SALEEM, KHANT NYI ZAYA, SAIFULLAH SYED, HUMAIRA JANANTH, MANAHIL ZARIEF

**Affiliations:** 1 Department of Internal Medicine, Akhtar Saeed Medical College/Farooq Hospital, Faisalabad, Pakistan; 1 Department of Internal Medicine, Akhtar Saeed Medical College/Farooq Hospital, Faisalabad, Pakistan; 2 Department of Internal Medicine, Watford General Hospital, West Herefordshire NHS Trust, West Herefordshire, United Kingdom; 2 Department of Internal Medicine, Watford General Hospital, West Herefordshire NHS Trust, West Herefordshire, United Kingdom; 3 Department of Internal Medicine, Riphah International University, Lahore, Pakistan; 3 Department of Internal Medicine, Riphah International University, Lahore, Pakistan; 4 Department of General Medicine, University of Algiers, Algiers, Algeria; 4 Department of General Medicine, University of Algiers, Algiers, Algeria; 5 Department of Internal Medicine, University Hospitals Leicester, Leicester, United Kingdom; 5 Department of Internal Medicine, University Hospitals Leicester, Leicester, United Kingdom; 6 Department of Internal Medicine, Evercare Hospital, Dhaka, Bangladesh; 6 Department of Internal Medicine, Evercare Hospital, Dhaka, Bangladesh; 7 Department of Internal Medicine, Al Aleem Medical College, Lahore, Pakistan; 7 Department of Internal Medicine, Al Aleem Medical College, Lahore, Pakistan; 8 Department of Internal Medicine, Velindre Cancer Centre, NHS Wales, Cardiff, Wales, United Kingdom; 8 Department of Internal Medicine, Velindre Cancer Centre, NHS Wales, Cardiff, Wales, United Kingdom; 9 Department of Vascular Surgery, Royal College of Surgeons in Ireland, Dublin, Ireland; 9 Department of Vascular Surgery, Royal College of Surgeons in Ireland, Dublin, Ireland; 10 Department of Cardiology, Evercare Hospital, Dhaka, Bangladesh; 10 Department of Cardiology, Evercare Hospital, Dhaka, Bangladesh

**Keywords:** MASLD, fatty liver index, cardiovascular risk, INTERHEART risk score

## Abstract

**Objectives:**

Metabolically associated steatotic liver disease (MASLD) is a growing health issue of concern and is frequently associated with cardiovascular risk. This study aimed to establish the association between MASLD and cardiovascular risk scores among urban adults.

**Materials and Methods:**

The study involved 300 adults recruited to participate in a cross-sectional survey at community health centres and outpatient clinics in different cities of Pakistan. A structured questionnaire was used to collect data, which included demographics, the Fatty Liver Index (FLI) to assess MASLD, and the INTERHEART Modifiable Risk Score (IHMRS) to evaluate cardiovascular risk. The statistical tests were conducted using SPSS v.26, which included descriptive statistics, Spearman correlations, the Mann-Whitney U-test, the Kruskal-Wallis test, the chi-square test, and multiple linear regression.

**Results:**

The majority of participants were men (N = 210, 70%), and the majority were between 40 and 49 years old (N = 90, 30%). FLI was positively associated with cardiovascular risk (r = 0.68, p < 0.001). Both FLI and IHMRS were much more common among men (p < 0.01). The prevalence of fatty liver and cardiovascular risk was highest in participants aged 40-49 years (p < 0.05). The results of triglycerides, GGT, BMI, and FLI (B = 0.065, p < 0.001) were found to be significant predictors via regression analysis.

**Conclusions:**

MASLD was strongly associated with cardiovascular risk, particularly in men and middle-aged adults. Incorporating liver health screening into cardiovascular screenings can aid in early detection and prevention.

## Introduction

### Background

Metabolically-dysfunctional-associated steatotic liver disease (MASLD), formerly non-alcoholic fatty liver disease, is the most common liver disorder globally. Its progressive type, metabolic dysfunction-related steatohepatitis (MASH), is connected with cardiovascular disease, sarcopenia, and chronic kidney disease by inflammatory spillover of lipotoxicity^1)^. It is the result of the ectopic accumulation of fats within hepatocytes, which can, under extreme circumstances, transform into metabolic-associated steatohepatitis (MASH), fibrosis, cirrhosis, or hepatocellular carcinoma. One of the mechanisms that leads to the progression of the disease is dysregulated lipid metabolism^2)^.

MASLD is a metabolic dysfunction-related disorder that occurs in more than one-third of the global adult population and is closely related to type 2 diabetes, insulin resistance, and obesity. It frequently accompanies atherosclerotic cardiovascular disease (CVD) and can play a role in cardiovascular events, making it critically important to provide an integrated cardiometabolic risk management^3, 4)^. The prevalence of MASLD is rising steadily worldwide, and it is more common in men and older adults. Concerningly, the incidences are also being reported in children, and projections in the future show that the number will increase many times, with the need to have preventive measures^5, 6)^.

Cardiovascular disease continues to be a significant health issue in the world, and proper identification and stratification of at-risk people are essential^7)^. MASLD is closely related to cardiometabolic risk factors, and CVD is the cause of death among patients^8)^.

In a study, MASLD was found among 36.4% and it significantly increased the risk of cardiovascular disease^9)^. Sex differences have a considerable effect on MASLD development and cardiovascular causes. Women are relatively safe, although hormonal changes like puberty, pregnancy, and menopause make women more susceptible. Knowing these differences is essential in the development of sex- specific preventive and therapeutic strategies^10)^.

### Study gap and objectives

The association of steatotic liver disease with metabolism is known as metabolically associated steatotic liver disease (MASLD), which is closely linked to cardiovascular disease in the global population. However, the majority of studies in this area have been conducted on populations with varied genetic and lifestyle backgrounds, unlike those in South Asia. Metabolic diseases like diabetes, obesity, and hypertension are becoming a common occurrence in Pakistan, but MASLD is little known. Since dietary, physical activity, and healthcare behaviour differ, the results of other international studies may not be fully applicable to Pakistani adults. The study aimed to investigate the prevalence of MASLD, assess cardiovascular risk using validated tools, and determine the strength of the association between MASLD and cardiovascular risk in urban adults to inform early detection and prevention strategies.

## Materials and Methods

### Research design

This was a cross-sectional study that sought to examine the relationship between Metabolically Dysregulated Steatotic Liver Disease (MASLD) and cardiovascular risk scores in urban adults. The design was selected in the study because it allows evaluating associations within a limited number of time and resources, which will provide a clear image of the relationship between MASLD and cardiovascular risk in this population. Although it is not possible to determine causality, the approach enables hypothesis testing and the generation of evidence to guide future studies. The participants were recruited from community health centres, outpatient clinics, and medical practices in urban Pakistan to ensure a diverse representation of individuals with varying socioeconomic and lifestyle statuses.

### Sampling strategy and population size

The World Health Organisation (WHO) formula was used to estimate the population proportion. A 95% confidence interval and a 5% margin of error were determined to select the sample. The lowest possible sample size was approximately 384 respondents, given a population proportion of 0.5 and accounting for possible non-responses^11)^. The limited time and resources were utilised to achieve the maximum possible sample size of 300, which was deemed sufficient to provide a statistically significant analysis and explore the interdependency between MASLD and cardiovascular risk scores. Convenience sampling was non-random. Every potential respondent who met the eligibility criteria and visited the community health centres, outpatient clinics, and medical practices of the sampled hospitals during the study period was invited to participate in the study.

### Inclusion and exclusion criteria

The participants were adults aged 18 years and older, residing in urban areas of Pakistan, who were willing to participate, provided informed consent, and were able to complete the questionnaire and undergo a basic clinical examination. Patients whose liver disease was not attributed to metabolic dysfunction, e.g., viral hepatitis, autoimmune hepatitis, or heavy alcohol consumption, were excluded so that only MASLD cases were factored in. Respondents who had been identified to have chronic diseases such as chronic kidney disease, active malignancy, or severe cardiac failure were also excluded, as this might confound the measurement of cardiovascular risk. Any incomplete questionnaires and lack of necessary clinical data were excluded from the final analysis.

### Data collection tools

Our structured questionnaire consisted of three major sections, including questions on the demographic data of the participants, measurement of fatty liver disease by the Fatty Liver Index (FLI), and measurement of cardiovascular risk through the INTERHEART Modifiable Risk Score (IHMRS). These instruments were selected due to their applicability and familiarity among urban populations in Pakistan. FLI and IHMRS were applied in the original English versions to guarantee accuracy and consistency.

### Demographic information

The demographic and background part of the questionnaire included age, gender, marital status, level of education, occupation, and other lifestyle factors such as smoking, physical activity, and food habits. This information was required for analysing the possible contribution of social and personal factors to the development of MASLD and cardiovascular risk among the study participants.

### Fatty liver index (FLI)

The Fatty Liver Index (FLI) was initially developed in 2006 by Bedogni et al. as a non-invasive algorithm to predict the presence of hepatic steatosis. It is based on four clinical and biochemical variables, including body mass index (BMI), waist circumference, triglycerides, and gamma-glutamyl transferase (GGT). It can be implemented in both research and clinical settings, as it consists of just four items. The FLI is calculated based on a logistic regression equation, which yields a score ranging from 0 to 100. A score of less than 30 has high sensitivity for excluding fatty liver, whereas a score of more than 60 indicates the presence of fatty liver with high specificity; a range between 30 and 60 indicates an indeterminate zone. This tool has demonstrated high internal consistency, with Cronbach's alpha values in validation studies reported to be higher than 0.80, indicating reliability in measuring the likelihood of MASLD. The original version of the FLI in English was utilised without modifications in this study, as it needs to be standardised^12)^. This is a tool licensed under the Creative Commons Attribution License (CC BY 2.0). Being a public domain open-access instrument, it can be freely utilised in research, and no further license is necessary, provided the original work is properly cited.

### INTERHEART Modifiable Risk Score (IHMRS)

In this research, we incorporated the INTERHEART Modifiable Risk Score, published in 2010 following the results of the original INTERHEART study by Yusuf et al. in 2004. This is a tool that assesses the risk of CVD development of a person by emphasising modifiable factors that include smoking, hypertension, diabetes, obesity, eating habits, physical exercise, psychosocial stress, and alcohol consumption. The INTERHEART score has been shown to perform well across a wide range of populations, including South Asians, consistently achieving a high value under the receiver operating characteristic curve (AUC) (above 0.75), which indicates excellent risk discrimination. It is a valuable tool for assessing the cardiovascular risk of the Pakistani population due to its simplicity, reliability, and applicability^13)^. The use of this tool was formally permitted through email correspondence with the developer.

### Study procedure

Participants were recruited between January 2025 and May 2025, after providing informed consent in community centres and at outpatient clinics. The respondents completed the questionnaire either independently or with the assistance of trained research staff, according to their preference and literacy level. In the case of the INTERHEART Modifiable Risk Score and Fatty Liver Index (FLI), data on lifestyle and demographic variables, such as smoking, diet, physical activity, and stress, were collected directly using a questionnaire. Nonetheless, some parameters were not self-reported (such as BMI, waist circumference, blood pressure, history of diabetes, triglyceride levels, and GGT) because they were collected from participants' medical records and laboratory reports. All responses were anonymous, and no personal information was associated with the data to maintain confidentiality.

### Data analysis

The data were analysed with the help of IBM SPSS Statistics version 26 (IBM Corp.). Descriptive statistics were used to summarise the demographics of the participants, and the results were reported in frequencies and percentages. The visual inspection of the main study variables used a detrended Q-Q plot to evaluate their normality. The Spearman correlation coefficient was used to test the correlation between the FLI and the IHMRS. Gender differences in the scores of FLI and IHMRS were examined using the Mann-Whitney U test, and differences between age groups were assessed using the Kruskal-Wallis test. To identify the predictors of IHMRS scores in relation to FLI, triglycerides, GGT, and BMI, multiple linear regression analysis was performed. In addition, the chi-square test was used to examine the relationship between gender and sex-specific categories of waist circumference. All the statistical tests were two-tailed and significant at p < 0.05.

### Ethical protocol

The study was conducted in accordance with the ethical principles governing research involving human subjects. The study was conducted in accordance with the Declaration of Helsinki and the principles of ethics governing research involving human subjects. The data collection had been pre-reviewed and approved by an Institutional Review Board (88s0-AAMCL-ERB-2025) of the Al-Aleem Medical College, Lahore. Ethical protection was provided through participant autonomy, maximisation of benefits, and minimisation of risks. The objectives, procedures, risks, and benefits of the study were explained in detail to all participants.

Participation was also voluntary, and respondents could withdraw at any time without facing any negative repercussions. Strict data confidentiality was ensured. No identifiable data were associated with the responses, and all data were stored on securely password-protected computers and in locked filing cabinets. Only the authorised personnel of the research team were allowed to access sensitive information in accordance with the IRB- approved data security procedures.

## Results

### Demographic profile of study participants

The demographic data for the 300 participants are presented in Table 1. The age group of 40-49 years (N = 90, 30%), males (N = 210, 70%), and married individuals (N = 130, 43%%) were the most prevalent age groups and gender, respectively. The majority were students (N = 139, 46%), primarily at the primary/middle school level (N = 103, 34%). In terms of lifestyle, 94 (31%) individuals never smoked, 135 (45%) were former smokers, and 71 (24%) were current smokers; the majority of participants reported moderate physical activity (N = 121, 40%). More than half stated that they had a family history of heart disease (N = 158, 53%), and frequently used drugs were those that lower blood pressure (N = 144, 48%) and those that control diabetes (N = 99, 33%). The majority of the participants had extremely high waist circumference (N = 180, 60%), high gamma-glutamyl transferase (N = 180, 60%), indeterminate fatty liver index (N = 190, 63%), borderline/high triglycerides (N = 165, 55%), and were overweight or obese (N = 260, 87%).

**Table 1:** Demographic characteristics of participants (N = 300)

Variable	f	%		Variable	f	%
Age				Moderate (≥ 150 min/week of moderate activity)	121	40
18-29 years	40	13		Vigorous (≥ 75 min/week of vigorous activity)	37	12
30-39 years	70	23		Family history of heart disease		
40-49 years	90	30		No	112	37
50-59 years	65	22		Yes	158	53
60 years and above	35	12		Don't know	30	10
Gender				Current medications		
Male	210	70		Blood pressure medications	144	48
Female	90	30		Diabetes medication	99	33
Marital status				Cholesterol medication	46	15
Single	92	31		None	7	3
Married	130	43		Other	4	1
Divorced/Separated	67	22		Waist circumference (sex-specific)		
Widowed	11	4		Normal	30	10
Educational level				Borderline	90	30
No formal education	83	28		Very high	180	60
Primary/Middle school r	103	34		FLI group		
High school/Secondary	77	25		Fatty liver unlikely (< 30)	12	4
Graduate/Bachelor's	23	8		Indeterminate (30-59)	190	63
Postgraduate/Higher	14	5		Fatty liver likely (≥ 60)	98	33
Occupation				Triglycerides group		
Student	139	46		Normal (< 150)	30	10
Employed	83	28		Borderline/High (150-250)	165	55
Unemployed/Homemaker	14	5		Very high (> 250)	105	35
Retired	64	21		Gamma-glutamyl group		
Smoking status				Low (< 20)	25	8
Never smoked	94	31		Moderate (20-60)	95	32
Former smoker	135	45		High (> 60)	180	60
Current smoker	71	24		BMI group		
Physical activity				Regular (18.5-24.9)	40	13
Sedentary (little or no activity)	41	14		Overweight (25-29.9)	100	34
Light (walks, minimal exercise)	101	34		Obese (≥30)	160	53

Note. f = frequency, % = percentage; FLI = fatty liver index; BMI = body mass index; Values are presented as N (%), N = 300; No statistical comparisons were performed for demographic variables in this table

### Normality assessment of cardiovascular risk scores

**Figure 1:**
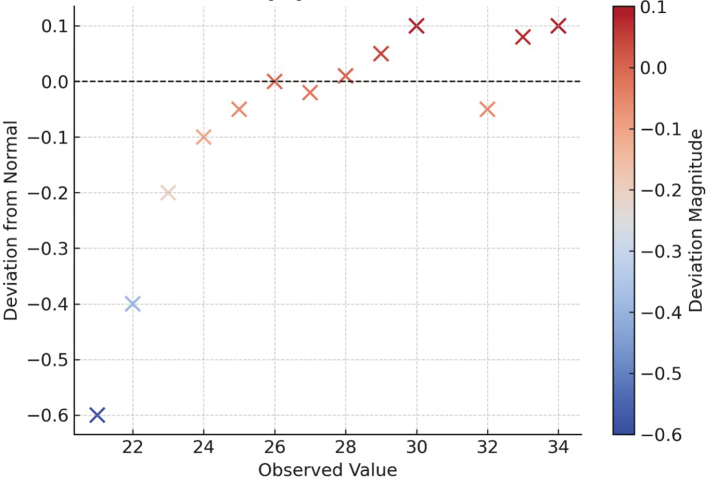
Detrended normal Q-Q plot of INTERHEART Risk Score Questionnaire total scores (N = 300)

Figure 1 gives a detrended normal Q-Q plot of total INTERHEART Risk Score Questionnaire scores on 300 participants. The plot evaluates the data normality, as it demonstrates that deviations from a perfectly normal distribution should be centred on the horizontal zero line. The points are concentrated around zero, indicating that the data are generally normally distributed, but there are deviations at the lower and higher observed values. The points in the cooler (blue) areas represent negative deviations, while the warmer (red) areas reflect positive deviations, with some notable outliers at the extremes. In general, the value indicates that the data of the INTERHEART Risk Score are relatively close to being normally distributed, but there is a slight non-normality in the tails.

### Correlation between MASLD and cardiovascular risk scores

A strong, positive, and statistically significant correlation between the FLI and the IHMRS (r = 0.68, t(298) = 16.01, p < 0.001) is shown in Table 2. This indicates that participants with high FLI values are more likely to score higher in cardiovascular risk, suggesting a strong correlation between the severity of fatty liver and the overall cardiovascular risk of the 300 participants in this study.

**Table 2:** Spearman correlations between fatty liver index (FLI) and INTERHEART risk score questionnaire (IHMRS) scores (N = 300)

Variables	Fatty Liver Index (FLI)	INTERHEART Risk Score Questionnaire (IHMRS)
Fatty Liver Index (FLI)	-	*r* = 0.68, *t*(298) = 16.01, *p* < 0.001**
INTERHEART Risk Score Questionnaire (IHMRS)	-	-

Note. All values are Spearman's rho (*r*) with corresponding *t*-statistics (*df* = 298) and two-tailed significance levels; a positive correlation indicates that increased FLI is associated with higher cardiovascular risk scores (IHMRS). *p* < 0.001 indicates statistical significance.

### Gender-based differences in liver and cardiovascular risk scores

Table 3 shows that the Fatty Liver Index (FLI) and INTERHEART Risk Score Questionnaire (IHMRS) scores were significantly different between men and women. In the case of FLI, the mean rank of men (N = 210) was 165.54, whereas that of women (N = 90) was 115.40, with a Mann-Whitney U of 6,291.00, a Z of -4.59, and a p-value of 0.001. Similarly, IHMRS scores were higher in men (mean rank = 162.80) than in women (mean rank = 121.80), U = 6,867.00, Z = -3.75, p < 0.001. These findings suggest that male participants of this sample had a higher level of fatty liver risk and cardiovascular risk than female participants.

**Table 3:** Mann-Whitney U test results comparing FLI and IHMRS scores between men and women (N = 300)

Outcome	Gender	N	Mean rank	Sum of ranks
Fatty liver index (FLI)	Male	210	165.54	34,764.00
	Female	90	115.40	10,386.00
INTERHEART risk score questionnaire (IHMRS)	Male	210	162.80	34,188.00
	Female	90	121.80	10,962.00
Test statistics	Mann-Whitney U	Wilcoxon W	Z	*p*
Fatty liver index (FLI)	6,291.000	10,386.000	-4.59	0.001**
INTERHEART risk score questionnaire (IHMRS)	6,867.000	10,890.000	-3.75	< 0.001**

Note. N = number of participants; Results from Mann-Whitney U tests comparing men and women on fatty liver index (FLI) and INTERHEART risk score questionnaire (IHMRS); Greater FLI scores reflect greater fatty liver risks, while higher IHMRS scores reflect higher cardiovascular risks; All comparisons were statistically significant at **p < 0.01.

### Age-related variations in liver and cardiovascular risk scores

The scores of the FLI and the IHMRS differed significantly among age groups, as shown in Table 4. The FLI scores also showed a significant increase between the ages of 18-29 years (mean rank = 120.50, N = 40) and 40-49 years (mean rank = 175.80, N = 90), and a slight decrease with the age of the respondents, χ^2^ (2(4) = 18.24, p = 0.001, r = 0.25. Likewise, the youngest group scored the lowest (mean rank = 125.10, N = 40), and the highest score was in the 40-49 years group (mean rank = 165.40, N = 90), F (2, 4) = 11.36, p = 0.023, r = 0.19. These results indicate that middle-aged participants had the most significant fatty liver and cardiovascular risk scores, with effect sizes suggesting small to moderate associations between age and both FLI and IHMRS scores.

**Table 4:** Kruskal-Wallis test results for fatty liver index (FLI) and INTERHEART risk score questionnaire (IHMRS) scores by age group (N = 300)

Outcome	Age group	N	Mean rank
Fatty liver index (FLI)	18-29 years	40	120.50
	30-39 years	70	160.20
	40-49 years	90	175.80
	50-59 years	65	155.30
	60 years and above	35	135.40
INTERHEART risk score questionnaire (IHMRS)	18-29 years	40	125.10
	30-39 years	70	150.70
	40-49 years	90	165.40
	50-59 years	65	155.10
	60 years and above	35	140.20
Test statistics	*x*^2^(df = 4)	*p*	*r*
Fatty liver index (FLI)	18.24	< 0.001**	0.25
INTERHEART risk score questionnaire (IHMRS)	11.36	0.023*	0.19

Note. N=number of participants; Results are from Kruskal-Wallis tests; Greater FLI scores reflect greater fatty liver risks, while higher IHMRS scores reflect higher cardiovascular risks; Effect sizes (*r*) were calculated as √χ^2^/N; **p < 0.001

### Predictors of cardiovascular risk among study participants

Table 5 indicates that the multiple regression model that predicts INTERHEART Risk Score (IHMRS) using Fatty Liver Index (FLI), triglycerides, gamma-glutamyl (GGT), and BMI was statistically significant, F (4, 295) = 38.10, p < 0.001, with 34% of the variance explained (R 2 = 0.34). Fatty Liver Index (B = 0.065, β = 0.310, t = 3.61, p < 0.001), triglycerides group (B = 2.450, β = 0.210, t = 2.78, p = 0.006), gamma-glutamyl group (B = 3.120, β = 0.185, t = 3.06, p = 0.002), and BMI group (B = 1.980, β = 0.240, t = 2.75, p = 0.007) were all significant positive predictors of cardiovascular risk. The findings indicate that increased FLI, triglycerides, GGT, and BMI are associated with higher IHMRS scores, suggesting the combined impact of these variables on the development of cardiovascular risk in the sample of 300 respondents.

**Table 5:** Multiple regression analysis predicting INTERHEART risk score (IHMRS) to fatty liver index (FLI), Triglycerides, Gamma-glutamyl (GGT), and BMI (N = 300)

Predictor	B	SE	β	t	*p*	95% CI LL	95% CI UL
Constant	18.20	5.90	-	3.08	0.002**	6.60	29.80
Fatty liver index (FLI)	0.065	0.018	.310	3.61	< 0.001**	0.030	0.100
Triglycerides group	2.450	0.880	.210	2.78	0.006**	0.72	4.18
Gamma-glutamyl group	3.120	1.020	.185	3.06	0.002**	1.11	5.13
Body mass index (BMI) group	1.980	0.720	.240	2.75	0.007**	0.56	3.40
Model summary	-	-	R	R2	Adjusted R2	F(4, 295)	p
Fatty liver index (FLI) - INTERHEART risk score questionnaire (IHMRS)	-	-	0.58	0.34	0.33	38.10	< 0.001**

Note. The regression model was statistically significant, explaining 34% of the variance in INTERHEART risk score (IHMRS). Higher fatty liver index, triglycerides, gamma-glutamyl transferase, and BMI were significant predictors of elevated cardiovascular risk; Unstandardized coefficients (B), standard errors (SE), standardized coefficients (β), t values, significance levels (p), and 95% confidence intervals (LL = lower limit; UL = upper limit) are reported; *p <0.05, **p <0.01, **p <0.001.

### Gender differences in central obesity indicators

Table 6 indicates a strong correlation between gender and sex-specific categories of waist circumference, χ^2^(2, 300) = 29.50, p < 0.001, with a moderate effect size (Cramer's V = 0.31). In male participants (N = 210), 25 (12%) had a normal waist circumference, 80 (38%) had a borderline waist circumference, and 105 (50%) had a very high waist circumference. Out of the women (N = 90), 5 (6%) were normal, 10 (11%) were borderline, and 75 (83%) had very high waist circumference. Such findings are an indicator that a larger proportion of women in this sample group had a very high waist circumference than men, a fact that emphasises the sex disparity in the risk of central obesity.

**Table 6:** Association between gender and sex-specific waist circumference categories (N = 300)

Gender	Normal (Men ≤94 cm; Women ≤80 cm); N (%)	Borderline (Men 95-102 cm; Women 81-88 cm); N (%)	Very high (Men ≥103 cm; Women ≥89 cm); N (%)	Total N(%)	*x*^2^ (df = 2)	*p*	Cramer's V
Male	25 (12%)	80 (38%)	105 (50%)	210 (70%)	-	-	-
Female	5 (6%)	10 (11%)	75 (83%)	90 (30%)	-	-	-
Total	30 (10%)	90 (30%)	180 (60%)	300 (100%)	29.50	<0.001**	0.31

Note. Frequency; % = percentage; df = degree of freedom; *x*^2^ = chi-square statistics; p = level of significance; p-values calculated using the chi-square test; χ^2^ (2, N = 300) = 29.50, *p* < 0.001.

## Discussion

This paper investigated the relationship between metabolically associated steatotic liver disease (MASLD) and cardiovascular risk in urban adults in Pakistan, using the Fatty Liver Index (FLI) and the INTERHEART Modifiable Risk Score (IHMRS). We found that the values of the Fatty Liver Index were highly correlated with the INTERHEART cardiovascular risk scores, which meant that MASLD is closely associated with cardiovascular risk. These findings align with an extensive population-based study, which concluded that increased FLI was associated with baseline cardiovascular risk factors as well as the subsequent incidence of cardiovascular disease^14)^.

In our study, the risk of fatty liver and CVD was greater in men as compared to women, as reflected by the higher scores in FLI and cardiovascular risk scores. This observation is consistent with previous research that shows that men are more likely to have NAFLD and suffer poor cardiometabolic phenotypes as well as a greater risk of cardiovascular problems than women^15)^.

In our study, the group aged 40-49 years showed the highest FLI scores, indicating that the risk of fatty liver is highest during middle age. This finding is consistent with previous research suggesting that metabolic risk factors play the most significant role in NAFLD development among individuals under the age of 65, particularly in the middle-aged population^16)^. On the same note, cardiovascular risk (IHMRS) was observed to be the most prominent amongst the 40-49 age group, and thus it can be said that midlife is a critical age group in terms of high risk of CV. This finding was consistent with previous research, which reported that middle-aged adults are at the highest overall cardiovascular risk^17)^.

Our multiple regression analysis revealed that the stronger FLI is associated with a higher cardiovascular risk (IHMRS), which aligns with previous evidence indicating that individuals with greater FLI not only have a higher baseline cardiovascular risk but are also at a greater risk of future cardiovascular events^14)^. Cardiovascular risk in our study was strongly associated with a high level of triglycerides, which goes in tandem with the epidemiological evidence that an augmented triglyceride level is a contributor to cardiovascular disease^18)^. We also established that higher GGT levels were a robust predictor of higher cardiovascular risk, which is consistent with previous research findings that GGT is associated with the presence of oxidative stress and metabolic dysfunction, as well as a higher risk of cardiovascular diseases^19)^. Furthermore, higher BMI in our study was strongly related to increased cardiovascular risk, which is in line with previous findings that obesity is a significant cause of cardiovascular disease and cardiovascular mortality^20)^.

In line with earlier findings, we observed substantial gender disparities in waist circumference (WC), with women comprising a larger percentage of the very high WC group than men, suggesting that sex-specific WC cut points are necessary^21)^.

The implications of the findings on the population health of Pakistan are enormous, as obesity, diabetes, and other metabolic disorders are becoming more common in the country rapidly. Considering the close correlation between MASLD and cardiovascular risk, liver dysfunction screening can be used as a predictive instrument of cardiometabolic disease. Early detection of those at risk might allow interventions such as lifestyle change, weight reduction, and more severe metabolic regulation to lessen the burden of the liver as well as cardiovascular disease in the long term.

### Study constraints

Several limitations of this research should be noted. Firstly, the cross-sectional design is unable to establish a causal relationship between MASLD and cardiovascular risk; longitudinal studies are necessary to verify temporal relationships. Second, there was no random sampling; therefore, the representativeness of the findings might have been low, as participants were recruited only from urban clinical and community settings. Third, the possibility of misclassifying MASLD severity by relying on diagnostic data based on the Fatty Liver Index (FLI) instead of imaging or biopsy may have contributed to the problem. Fourth, the INTERHEART Modifiable Risk Score, though tested in South Asian populations, is more of a reflection of modifiable risk and might not be a good measure of genetic or unmeasured environmental factors. Lastly, self-reported lifestyle information is subject to recall and reporting bias, which could have affected the quality of the findings.

### Implications for future research

A prospective cohort design should be used in future studies to determine the causal relationship between MASLD and cardiovascular outcomes in South Asian populations. Future research should include larger, multicenter studies in both rural and urban settings to enhance generalizability and consider regional differences in dietary and lifestyle patterns. Making the MASLD assessment more accurate would be possible by incorporating newer diagnostic tools, such as imaging, elastography, or serum biomarkers. Furthermore, interventional studies analysing the effects of weight loss, dietary alterations, and pharmacological interventions on MASLD cardiovascular outcomes would be a step towards viable evidence of preventive interventions. Finally, genetic, hormonal, and cultural factors, along with their implications for the occurrence of sex differences related to MASLD and the risk of cardiovascular diseases, can also be examined. This would aid in customising prevention and management efforts.

## Conclusion

This study suggests that the relationship between cardiovascular risk and MASLD is substantial and statistically highly significant among the adult urban Pakistani population. The levels of FLI, triglycerides, GGT, and BMI were high. Thus, they became key predictors of cardiovascular risk, confirming that liver and heart health are closely intertwined concepts. The risk affected men and middle-aged adults in particular, rendering the need for early diagnosis and sex-specific solutions. These findings suggest that cardiovascular screening programs should consider liver health tests as an intervention to raise awareness of cardiometabolic disease in high-risk groups. Improvement of policies that regulate the health of the masses in the prevention of obesity and sedentary lifestyles will be fundamental in minimizing the dual burden of both MASLD and cardiovascular disease in Pakistan.

## Author contributions

FH and KKN conceived and designed the study. FI and SO contributed to the study design and supervised data collection. MHN, SA, and MS were responsible for data acquisition and data entry. KNZ and SS conducted statistical analyses and interpreted the data. HJ assisted in manuscript drafting and reference organization. MZ served as the corresponding author, coordinated communication, and finalized the manuscript submission. FH and KKN critically revised the manuscript for important intellectual content. All authors read and approved the final version of the manuscript.

## Conflicts of interest statement

The authors declare that there are no conflicts of interest.
